# Fatigue Lifetime of Ceramic Matrix Composites at Intermediate Temperature by Acoustic Emission

**DOI:** 10.3390/ma10060658

**Published:** 2017-06-16

**Authors:** Elie Racle, Nathalie Godin, Pascal Reynaud, Gilbert Fantozzi

**Affiliations:** University of Lyon, INSA de Lyon, MATEIS, UMR 5510, France; elie.racle@gmail.com (E.R.); pascal.reynaud@insa-lyon.fr (P.R.); gilbert.fantozzi@insa-lyon.fr (G.F.)

**Keywords:** ceramic matrix composite, acoustic emission, fatigue lifetime

## Abstract

The fatigue behavior of a Ceramic Matrix Composite (CMC) at intermediate temperature under air is investigated. Because of the low density and the high tensile strength of CMC, they offer a good technical solution to design aeronautical structural components. The aim of the present study is to compare the behavior of this composite under static and cyclic loading. Comparison between incremental static and cyclic tests shows that cyclic loading with an amplitude higher than 30% of the ultimate tensile strength has significant effects on damage and material lifetimes. In order to evaluate the remaining lifetime, several damage indicators, mainly based on the investigation of the liberated energy, are introduced. These indicators highlight critical times or characteristic times, allowing an evaluation of the remaining lifetime. A link is established with the characteristic time around 25% of the total test duration and the beginning of the matrix cracking during cyclic fatigue.

## 1. Introduction

Ceramic matrix composites are very interesting structural materials for high temperature applications. Even if the constituent materials are brittle, the strain at failure is rather high due to important matrix cracking and the deflection of cracks at interfaces [1]. Indeed, the fracture strain of the fibers is higher than fracture strain of the matrix. Then, when a load is applied to a composite, the matrix cracks first and the fibers bridging this crack sustain the load. Due to the difference of stresses between bridging fibers and the matrix at the level of the matrix crack, interface between fiber and matrix is subjected to a shear stress, leading to a debonding of fibers and matrix [2]. This material behavior is affected by oxidation of interphases and fibers and the ultimate failure is governed by slow crack growth in the fibers [3]. Self-healing material has been developed [4,5] to protect fibers against oxidation, which greatly increased the material lifetime. Nevertheless, under air and for temperatures below 550 °C, self-healing is not important enough to fully protect the material. 

CMCs will be used in the new generations of civil aircraft engines owing to their low density and good mechanical properties. These applications require a very long lifetime in service conditions. Therefore, a significant issue is a better understanding of the damage mechanisms and kinetics, in order to perform reliable estimations of material lifetime. 

Various authors have studied CMC mechanical behavior and degradation mechanisms at high temperatures [3,6,7,8]. Many studies have attempted to understand the links between microstructure, damage and durability of these materials [9,10,11,12,13,14]. Now more information is needed at intermediate temperatures: the oxidation kinetics of the different constituents are complex, and the effect of matrix sealing on the lifetime of the composite has to be examined. Today, the challenge is predicting components’ lifetime in service. The lifetimes under cyclic and static fatigue are rather long, which make it hard to realize tests on laboratory equipment. This kind of study needs to be done with a limited number of tests, thus the use of different techniques to monitor the damage in real time is almost mandatory.

To achieve this goal, quantification of damage, as well as identification of the various damage modes, are required. Acoustic emission (AE) appears to be a good candidate in this case [15]. AE is a technique used to detect in-situ information about damage that occurs during long term mechanical tests. In the case of composite materials, many mechanisms have been confirmed as AE sources including matrix cracking, fiber-matrix, interface debonding, fiber fracture and delamination. AE consists of recording transient elastic waves on the material created by damage mechanisms. There are several studies referring to this kind of method for different types of CMCs [16,17,18,19].

A main purpose of this paper is to perform deeper analysis for predicting the remaining lifetime from damage evolution recorded by AE technique. The study consists of analyzing and comparing material behavior under cyclic and static fatigue loadings at the same temperature and under air, in order to determine the effects of cyclic loading on damage and lifetime. For fatigue tests, AE activity can be analyzed from different points of view. First, by linking each acoustical event to the damage mechanism that generated it. This process needs clustering algorithms [20]. In this way, a careful analysis of acoustic emission signals may lead to the discrimination of the different damage mechanisms occurring in a composite material. This is a possible solution for the identification of damage during service, with a view to component lifetime control. Another approach based on a global AE analysis consists of considering the evolution of released energy [21,22,23] with a view to identify a critical point.

It is generally accepted that the energy of an AE signal is related to the energy released by the source. Consequently, AE energy gives information about material damage; it is then possible to point out precursory elements to ultimate failure or to simulate AE energy evolution with a power law, to determine lifetime. 

In this study, in order to predict the remaining useful lifetimes during fatigue tests, new indicators of damage have been defined, based mainly on acoustic energy analysis. Afterwards, some new procedures based on the AE are presented for the identification of critical times. The results indicate that the AE method could be more suitable than conventional methods to reveal critical times. Indeed, these indicators highlight critical times or characteristic times, allowing an evaluation of the remaining lifetime around 25% of the total test duration during cyclic fatigue tests. Moreover, the clustering of acoustic emission, using a supervised clustering method based on random forest approach [24], makes possible to get a real-time detection of each damage phenomena and to identify the mechanism responsible for this critical time.

The paper is organized as follows: Section 2 resumes the experimental procedure, Section 3 presents the definition for the damage indicators, Section 4 is dedicated to the classification of the AE signals with a supervised clustering and Section 5 discusses the results. At first, damages are investigated during incremental static fatigue test and incremental cyclic fatigue tests at high temperature by the AE method. Afterwards, based on the experimental results, procedures for predicting the remaining lifetime are carried out on cyclic fatigue tests at constant maximum amplitude. 

## 2. Experimental Procedure

### 2.1. Materials and Mechanical Tests

The composite studied consists of a self-healing [Si-B-C] matrix reinforced with Nicalon SiC fibers, coated with PyC and is manufactured by SAFRAN Ceramic group (Bordeaux, France) [1]. The reinforcement architecture is a stack of several layers of 2D satin fabrics linked together by strands of fibers in the third direction. In this study, all the specimens have a dog-bone shape with a thickness of 4 mm and a gauge section of 60 mm × 16 mm. Fatigue tests have been realized at a temperature of 450 °C under air, because at this temperature the material is not protected by self-healing.

For static fatigue tests, specimens are first loaded at a constant loading rate of 1 kN/min, and periodically (every 6 or 12 h) unloading-reloading cycles are carried out in order to determine the secant elastic modulus. 

Cyclic fatigue tests have been conducted under a tensile/tensile sinusoidal loading with constant amplitude and a frequency of 2 Hz (*R* = 0). Fatigue tests are performed by applying a maximum load calculated as a percentage of the ultimate strength. Incremental fatigue tests have been realized, where several loadings ranging from 0.18 to 0.84 UTS (Ultimate Tensile Strength) have been applied. The UTS is obtained from quasi-static tensile test. Three samples were tested for each condition. 

**Figure 1:**
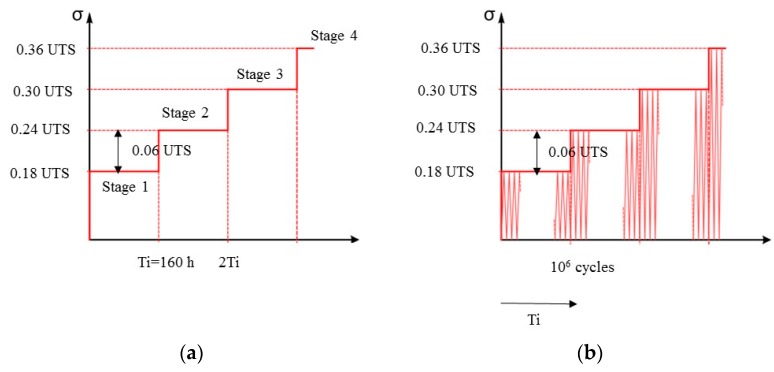
Applied stress for (**a**) Static fatigue test and (**b**) Cyclic fatigue test for four stages.

In the case of static fatigue tests, the imposed stress increases every Ti (160 h) with a step corresponding to 6% of the tensile strength. Cyclic fatigue tests have been realized with imposed stress oscillating at a frequency of 2 Hz between 0 and a constant maximum value which is incremented of 6% every 10^6^ cycles (Figure 1).

For all tests, a specimen is heated to the testing temperature and held at temperature for 30 min prior to testing. A high temperature extensometer on the gauge length section of the specimen is used during each tests. Potential indicators of damage evolution are obtained by recording the secant modulus and the maximal strain. To characterize the dissipation of energy during a loading/unloading cycle, several parameters may be calculated as the area of the stress/strain loop ∆W, the internal friction ∆W/We (where We is the maximum elastic energy stored during the cycle) [2].

### 2.2. Acoustic Emission Monitoring

During tests, AE is recorded with two piezoelectric sensors (Micro80, Mistras Group, Paris, France) fixed on the specimen surface. A medium viscosity vacuum grease is used as coupling between the specimen’s surface and sensors. Each sensor is connected to the data acquisition system via a preamplifier with a 40 dB gain and 20–1200 kHz bandwidth. The input parameters used for AE monitoring are as follows: peak definition time (PDT = 20 μs), hit definition time (HDT = 40 μs) and the hit lockout time (HLT = 1000 μs). An acoustic threshold level at 34 dB for the static fatigue test or at 46 dB for the cyclic fatigue is used to eliminate background noise. Previous works have shown that no damage occurs below 46 dB during tensile tests [17].

The position of a source detected can be determined linearly knowing the wave velocity in the material. The location of AE sources is determined from the difference in arrival times at the sensors. 

The AE wave velocity has been calibrated before the test and noted *Ce*_0_. Pencil lead break procedure is used to generate repeatable AE signals for the determination of the wave velocity. The velocity *Ce*(*ε*) of an extensional wave in a thin plate is proportional to the square root of the elastic modulus E of the material. Since E decreases as damage occurs in the material, it is important to take into account the evolution of Ce during the mechanical test in order to better evaluate the location of the AE sources. As proposed by Morsher [25], the initial modulus during unloading *E*(*ε*) is measured during a cycled tensile test, where hysteresis loops are obtained at different strains. The velocity *Ce*(*ε*) is then determined by using Equation (1):

(1) $$\frac{Ce(\varepsilon )}{Ce_{0}}=\sqrt{\frac{E(\varepsilon )}{E_{0}}}$$

where *C_e_*_0_ and *E*_0_ are respectively the velocity and the elastic modulus in the undamaged state, *Ce*(*ε*) and *E*(*ε*) are respectively the velocity and the elastic modulus under a strain *ε*. Just prior to the final rupture, the velocity on the composite is found to be equal to 6480 m/s, instead of 10,000 m/s in the undamaged state. Thus, the decrease in wave velocity is not negligible. 

On each waveform, twenty-one AE parameters, in time and frequency domains, are recorded or calculated for all the specimens (Table 1).

## 3. Damage Indicators

The definition of the damage indicators is based on the determination and on the investigation of the acoustic energy released. Each AE source n is described by the time t at which the closest sensor is triggered and the amount of energy recorded by the sensors *E*_1_(*n*) and *E*_2_(*n*). The source energy is then defined as the square root of the product of the amounts of energy received at both sensors for each source.

(2) $$E\left(n\right)=\sqrt{E_{1}\left(n\right)\times E_{2}\left(n\right)}$$

AE data and mechanical information can be analyzed independently. However, combination of mechanical energy (*U_m_*) and acoustic energy (*U_AE_*) denoted Sentry Function (*F*), initially introduced by Minak [26,27], can be used in order to obtain a better damage characterization:

(3) $$F=ln\frac{U_{m}\left(x\right)}{U_{AE}\left(x\right)}$$

where *U_m_*(*x*), *U_AE_*(*x*) and *x* are the strain energy, the cumulated acoustical energy and the strain of the tested material respectively. It is defined as soon as the first acoustical event is recorded. Strain energy is calculated by measuring the area under the force-displacement curve. To determine only the effects of tension, unloading/loading loads are not taken into account. This global function is calculated for every k acoustic source (k ~ 0.1% of number of signals). Sudden drops of the function may be related to the occurrence of significant internal material damage.

The coefficient of emission [21] *R_AE_* is defined as the increment of energy ∆*E* recorded during an increment of time ∆*t*:

(4) $$R_{AE}=\frac{∆E}{∆t}$$

∆*E* is the cumulative AE energy for all signals recorded during the interval [*t*; *t* + ∆*t*]. This rate is calculated every n signals. n is chosen depending on the acoustic activity (n ~ 1% of number of signals). It has to be high enough to get a smooth curve. 

**Figure 2:**
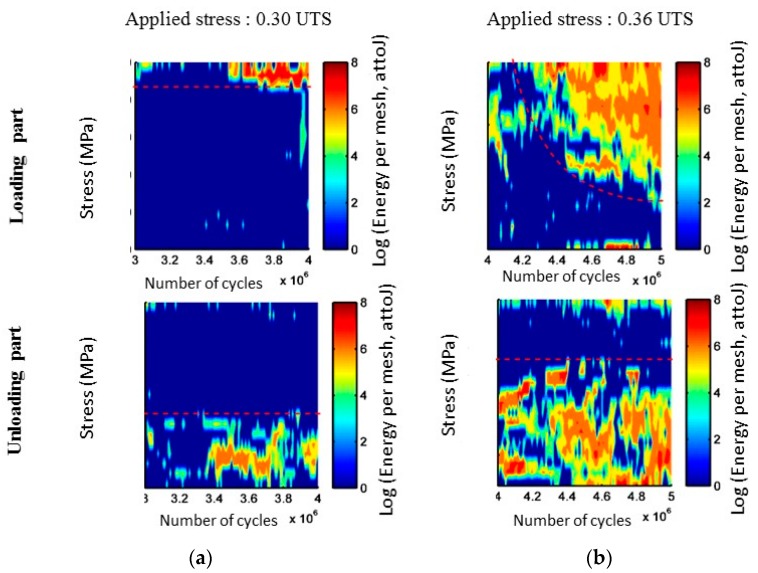
Evolution with the number of cycles of the linear density of acoustic energy recorded during loading part and unloading part of the cycles on SiC_f_/[Si-B-C] composites for two different applied loads (**a**) *σ* = 0.30 UTS and (**b**) *σ* = 0.36 UTS.

During cyclic fatigue tests, another indicator denoted *R*_LU_, is defined by the ratio of the cumulated liberated energy during the loading part of the cycles and the energy recorded during the unloading part. Figure 2a,b shows the evolution with the number of cycles of the linear density of acoustic energy recorded during loading part on one hand and unloading part of the cycles on the other hand. It is a mapping of the applied load during the detection of AE signals. This representation highlights different activity during these two steps. Moreover, the acoustic emission activity evolves with the applied stress.

(5) $$R_{LU}=\frac{\sum \text{​}E_{loading}}{\sum \text{​}E_{unloading}}$$

## 4. Damage Identification with a Random Forest Algorithm

A supervised classification technique may also be used to analyse AE signals recorded during fatigue tests of CMC composites. This technique requires a data base of signals that have been labelled: the training set. This training set is created by merging data collected during tensile test. As described in a previous paper [17], the analysis of AE signals, observation of microstructures and analysis of the mechanical behaviour of the composite led to the identification of 4 types of AE signals and to the following labelling of classes:

Class A: cluster A contains signals from two damage mechanisms which are chronologically well separated: seal coat cracking and tow breaks;
Class B: cluster B contains signals from two damage mechanisms which are chronologically well separated: longitudinal matrix cracking and individual fibre breaks in the fracture zone just before failure;
Class C: cluster C contains signals with relatively short duration, short rise time and low amplitude compared to the others: transversal matrix cracking;
Class D: this cluster is the last one to be activated and it becomes more active as strain increases and the D-type signals have a longer rise time compared to other signals: sliding at fibre/matrix interfaces, fibre/matrix debonding.

It seems difficult to isolate the fiber fracture from the remainder of the AE data, because the number of such fractures is very small in comparison with the global number of cracks. An additional class labelled E is introduced into the library in order to include the friction for the cyclic fatigue tests. The characteristics of AE signals recorded during the unloading part of the cycle are used as a reference to identify friction during loading. It corresponds to signals recorded under fatigue, during unloading steps of cycles after the cycle 2000 and for applied stresses lower than *σ*_max_/2–10 MPa. These signals are associated with the friction generated during cyclic fatigue. 

In order to establish the training set of labelled signals for the supervised analysis, the same amount of signals (2000 signals) is used in each class (A, B, C, D and E). This training set included all the damage modes that may operate in this composite. The supervised classification is based on a random forest algorithm [24]. During the testing phase, each AE signal runs down each tree of the forest, leading to T votes. The final decision can be obtained by two different ways. The first one is simply the usual majority voting (MV) rule. In this work, a second decision rule is introduced called the security voting (SV) rule. In this special rule, a given AE signal is assigned to a specific class if more than 51% of the total number of trees voted for that class and the second most represented class has to gather no more than 30% of the votes. For cyclic fatigue tests with constant maximal amplitude, between 47% and 62% of the classified signals validate these two criteria. For these tests, only the signals recorded during the loading part of the cycles are classified in order to minimize the friction emissions. 

## 5. Results and Discussion

### 5.1. Incremental Static Fatigue Tests and Incremental Fatigue Tests

**Figure 3:**
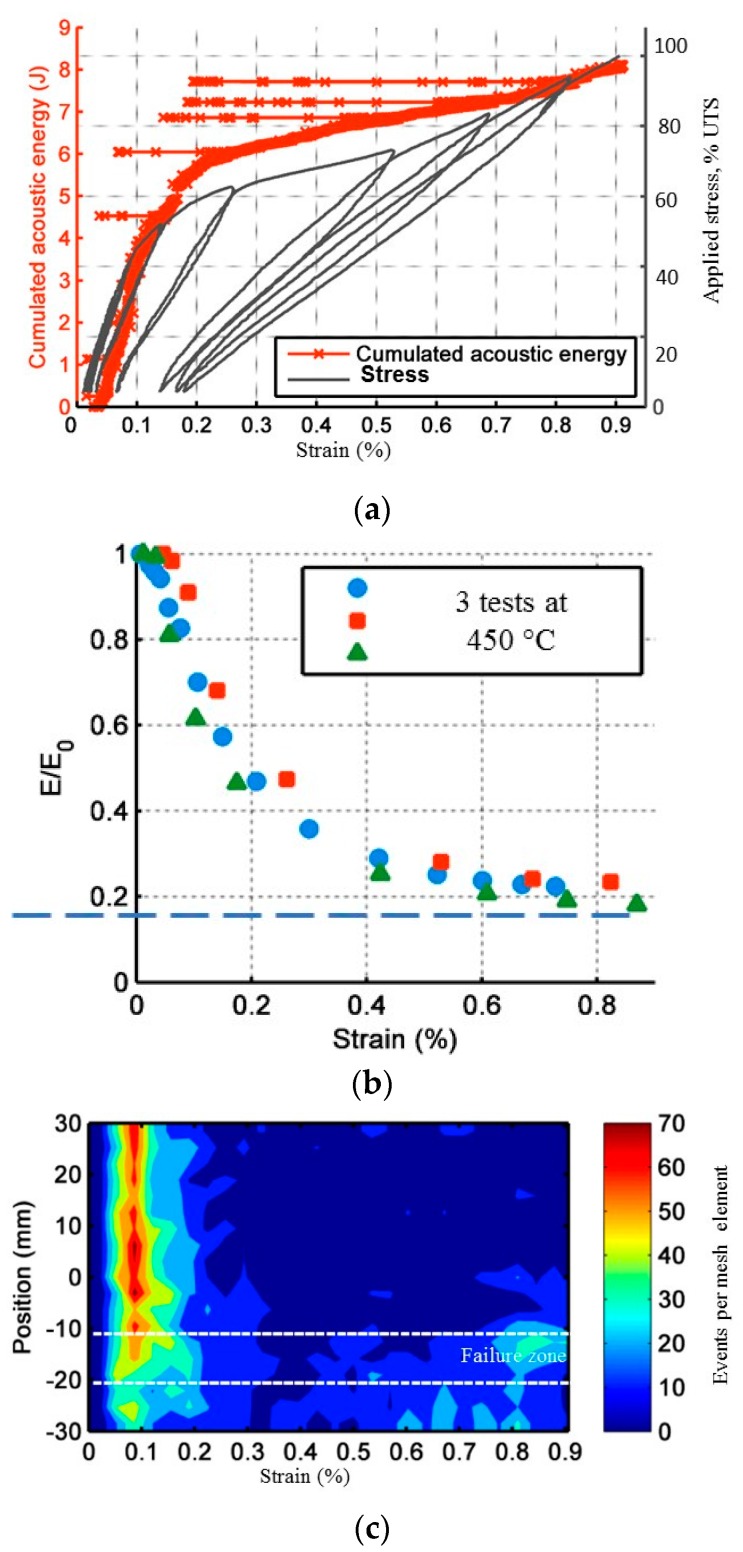
(**a**) Experimental mechanical curve of composite under monotonic tension at room temperature; (**b**) Evolution of elastic modulus for three tests; (**c**) Evolution of linear densities of acoustic events along the specimen axis during tensile tests at room temperature.

Results of incremental fatigue tests are summarized in Table 2. To compare the results of static and cyclic fatigue tests, the behavior of the material during a tensile test is used as a reference. Thereafter, *ε*_R_ will represent ultimate tensile strength and strain at failure respectively. During a tensile test (Figure 3), the mechanical behavior is composed of three distinct phases. For strain values lower than 0.1% specimens exhibit a linear behavior. Between 0.1% and 0.6% a significant decrease in elastic modulus is observed due to matrix cracking. Above 0.6%, the elastic modulus stabilizes indicating the saturation of matrix cracking. Beyond this point, the applied load is borne by fibers only. The cumulative number of AE signals allows the identification of the same phases. AE becomes significant in terms of the number of signals and energy beyond 0.1%. At 0.6%, a significant change is observed on the cumulative number of acoustic emission sources. This characteristic point is in very good agreement with the saturation of matrix cracking. Beyond this point, AE is lower and becomes very localized inside in the rupture zone. The analysis of the evolution of acoustic energy during a tensile test shows that signals with high energy are generated when matrix is cracking and at the end of the test when the whole load is carried by the fibers only, which break progressively (Figure 3). 

For both types of loading, stress at failure is lower than the ultimate tensile strength, nevertheless it is twice as high for static loading than for cyclic loading, which means that cyclic loading has an effect on the material lifetime. Considering the strain at failure, the value is also lower for a cyclic loading than for a static loading. In addition, the elastic modulus decreases greatly before ultimate fracture of the composite while it tends to an asymptotic value for a static loading, and does not reach the limit value Ef.vf for the cyclic loading. Moreover, the mean distance between matrix cracks in the longitudinal yarns is higher under cyclic fatigue, showing that saturation of matrix cracks could occur at a lower interfacial shear stress, decreased probably by progressive wear induced by cyclic loading. 

**Figure 4:**
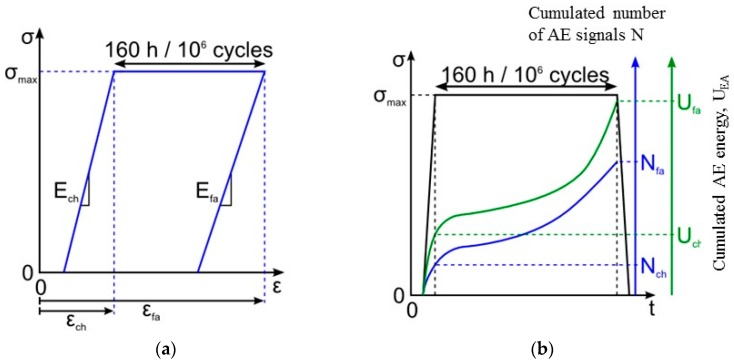
Schematic diagram: (**a**) elastic modulus determination for the first loading (*E*_ch_) of the stage and at the end of the stage (*E*_fa_) and (**b**) cumulated number and energy of acoustic emission signals calculated at the end of the first loading (*N*_ch_ and *U*_ch_) and at the end of the stage (*N*_fa_ and *U*_fa_).

**Figure 5:**
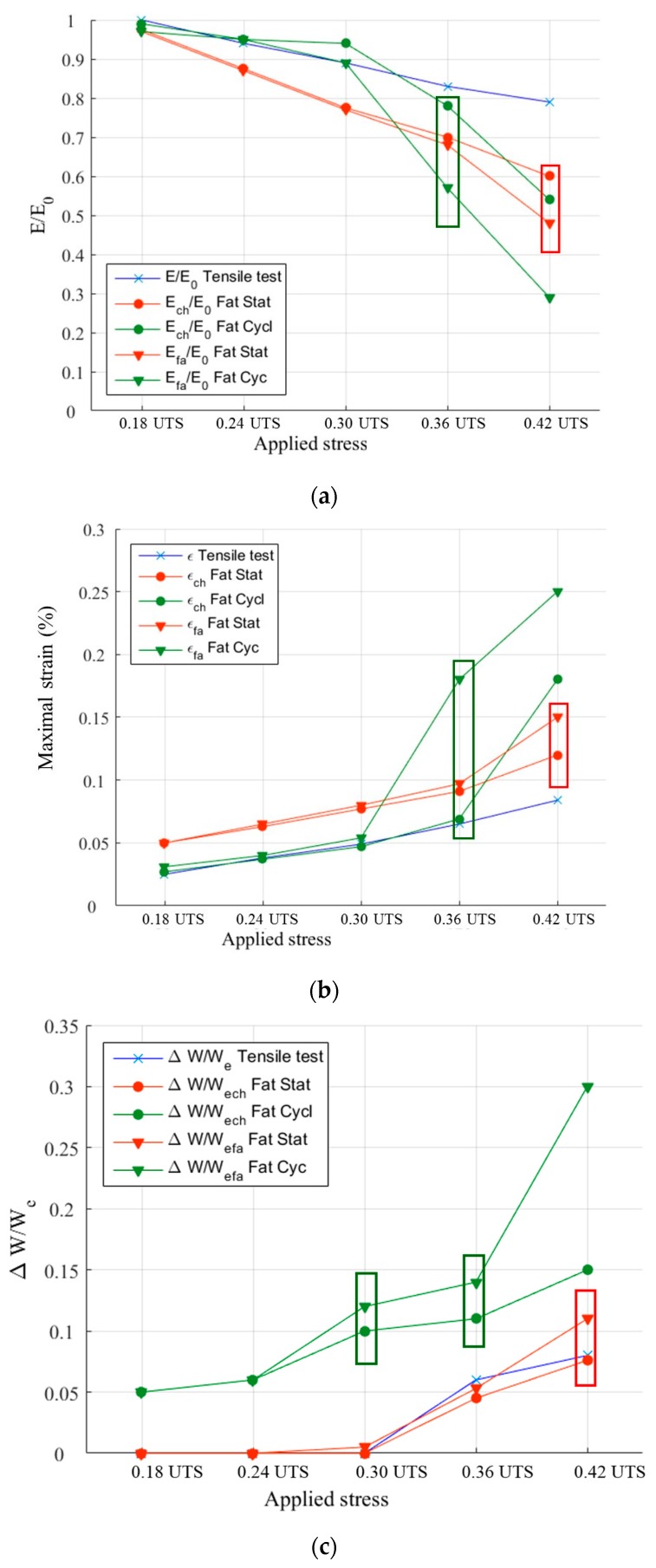
Evolution versus the applied load of (**a**) ratio *E*/*E*_0_; (**b**) maximal strain and (**c**) internal friction. The index “ch” means the first loading of each stage and the index “fa”, the end of the stage.

To display these differences, the mechanical parameters and the acoustic activity are compared, for every level of loading. The following ratios are calculated: *E*/*E*_0_ with E the secant elastic modulus and *E*_0_ the initial elastic modulus, N/N_Rt_ with N the number of recorded signals and N_Rt_ the number of signals recorded during the tensile tests, U_EA_/U_EART_ with U_EA_ the cumulated recorded acoustic energy and U_EARt_ the acoustic energy recorded during a tensile test. To estimate the effects due to fatigue, these parameters are calculated during the first loading of each stage or level of loading (identified by the index ch) and at the end of each stage (identified by the index fa) as presented in Figure 4. The comparison is made until a load level of 0.42 UTS. Moreover, Figure 5b shows the evolution of the strain during incremental fatigue tests, the strain at the beginning of the stage and the recorded strain at the end of the stage versus the maximum applied load are mentioned. On this figure, the strain recorded during a tensile test is also reported. Figure 5c represents the evolution of internal friction.

The comparison of elastic modulus, maximal strain and internal friction at the beginning and at the end of each level of loading (Figure 5) shows that for low levels of load (<0.3 UTS), the damage of the material remains close in tensile tests and in cyclic or static fatigue tests. Indeed, the decrease of the modulus or the increase of the maximal strain are only linked to the increase of the applied load. There is no evolution of these parameters during a stage. There is either no evolution of the internal friction between the first and the last cycle. However, the values calculated in the case of the cyclic fatigue are higher than under statique fatigue. The main effect of damage on mechanical parameters is on the variation of the internal friction ∆W/We at 0.30 UTS. From 0.36 UTS, there is an evolution of the damage during the stage. The load thus has an effect on the behavior of the material. The decrease of the secant modulus does not stabilize at this level of loading during the stage. 

**Figure 6:**
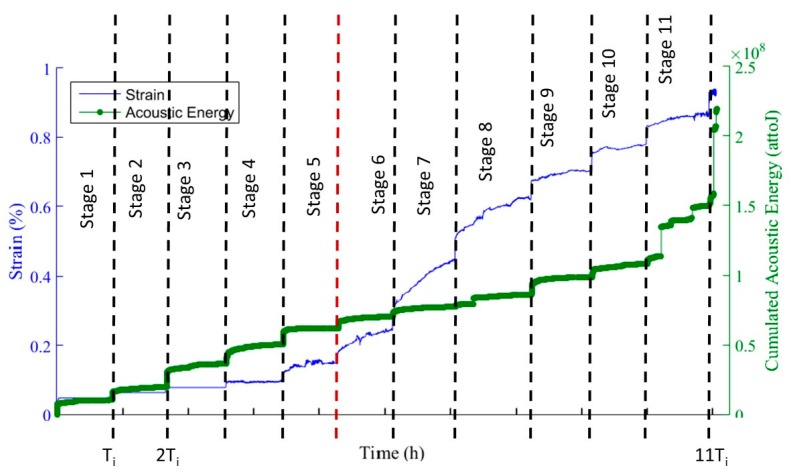
Strain and cumulated AE energy vs. time during a static fatigue test (stage 1: 0.18 UTS, stage 12: 0.84 UTS).

Analysis of acoustic energy during fatigue tests shows different behaviors depending on the type of loading (Figure 6). For a static loading, during each stages, the activity may be shared in 2 parts: AE generated during the loading part of the stage and AE generated when the load is steady. This makes it possible to distinguish 2 phases during the whole test (Figure 6). Within stages 1 to 5 (0.18 UTS to 0.42 UTS), the acoustic energy is more important during the loading part than when the load is constant (twice as high during the loading phase as during the constant load phase). It is the result of an important energy release when the matrix is cracking, whereas acoustic activity is weak at constant load, where the material is not damaged. Starting from stage 6 (0.48 UTS) the trend is reversed, constant load phases are more energy-generating. This shows that there is a transition in the damage mode between stages 5 and 6, and loading higher than 48% of UTS for a static fatigue test has consequences on material damage.

**Figure 7:**
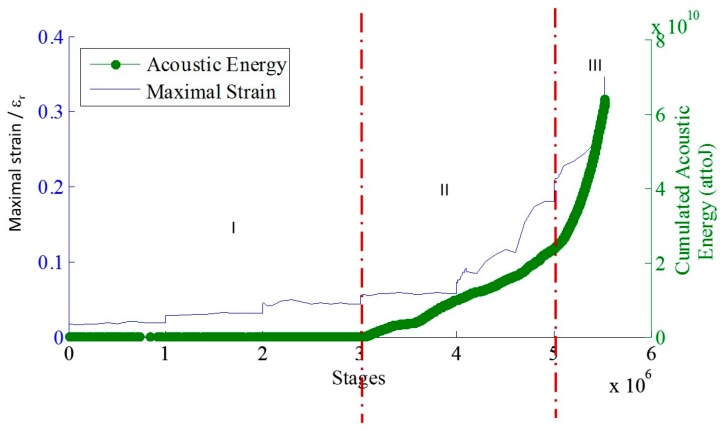
Maximal strain and cumulated AE energy vs. number of cycles during a cyclic fatigue test (stage 1: 0.18 UTS, stage 5: 0.42 UTS).

During cyclic fatigue tests, 3 parts clearly appear in the acoustic energy evolution (Figure 7). Part I until stage 2 (0.18 UTS to 0.24 UTS) is composed of approximately 9000 sources localized in the gauge section, whereas the average activity is 1 source per cycle in part II and III. This activity is generated mostly at the beginning of the stage, at maximal stress, which seems to be generated by matrix cracking. The important increase during part II shows that the material behavior is different when the load is cyclic already during stage 3 (0.30 UTS). It is generated mostly for maximal stress, but activity appears as well during the unloading part of cycles, for a low stress. This acoustic activity may be generated by friction at the interfaces when matrix cracks are closing and opening. A third part appears at stage 5 (0.42 UTS), which has the same average sources per cycles but a higher average energy per signals and can be associated with fiber breaks. Furthermore, even if the material does not seem to damage at stage 3 (0.30 UTS), from a mechanical point of view the different evolution of acoustic activity at that stage shows that there are already consequences of a cyclic load, which is probably due to wear at interfaces which could reduce the material lifetime [2].

**Figure 8:**
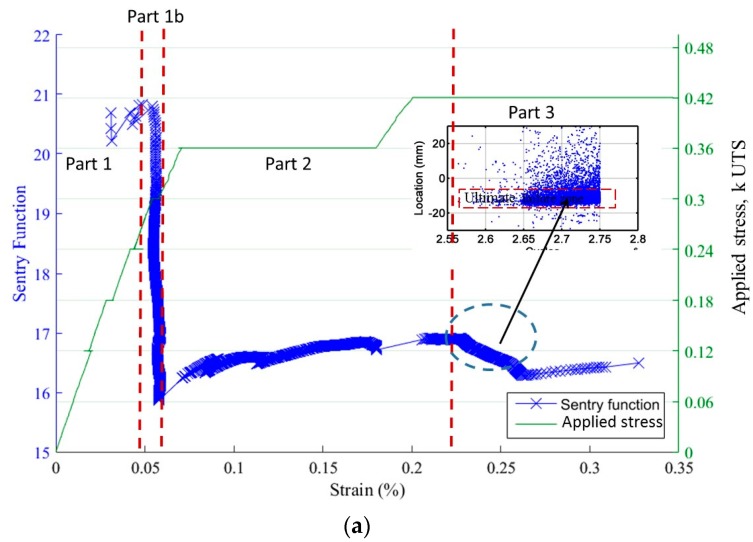

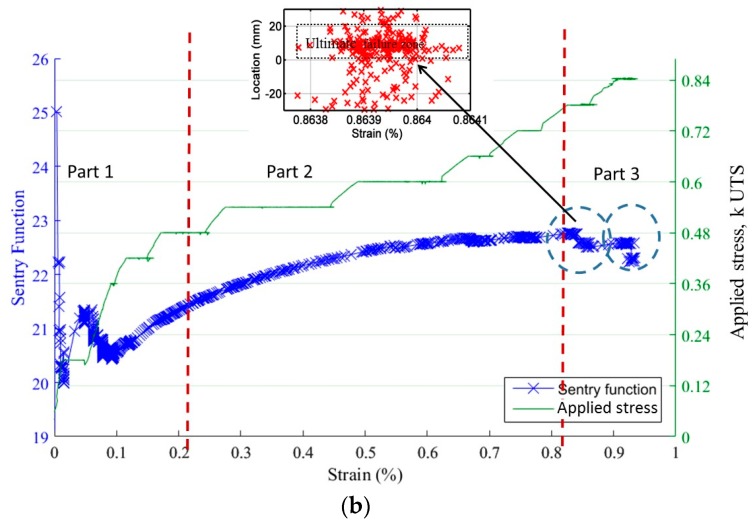
Coupling of mechanical energy and acoustic energy (Sentry function) for (**a**) an incremental cyclic fatigue test and (**b**) an incremental static fatigue test.

Figure 8a,b gives the evolution of the Sentry function (F) during static and cyclic loading. For both cases, a third part appears for an average time of 90% of the total test duration. The function is then decreasing. The AE signals which have created this decrease are probably generated by fiber breakages, since they are localized in the area where the specimen failed. The evolution of the Sentry function during the cyclic fatigue test shows an extra part (part 1.b) where, importantly, the function is decreasing. This is caused by the important increase of acoustic energy, whereas the strain remains constant. It appears that cyclic loading generates various damage mechanisms since the function does not show this type of variation for a static loading. The variations of this function in different cases exhibit several levels of damage on the composite: matrix cracking and fibers breakages for fatigue tests. Furthermore the Sentry function shows that a cyclic loading has more critical damage effect than a static loading when the maximal stress is higher than 30% of UTS. 

**Figure 9:**
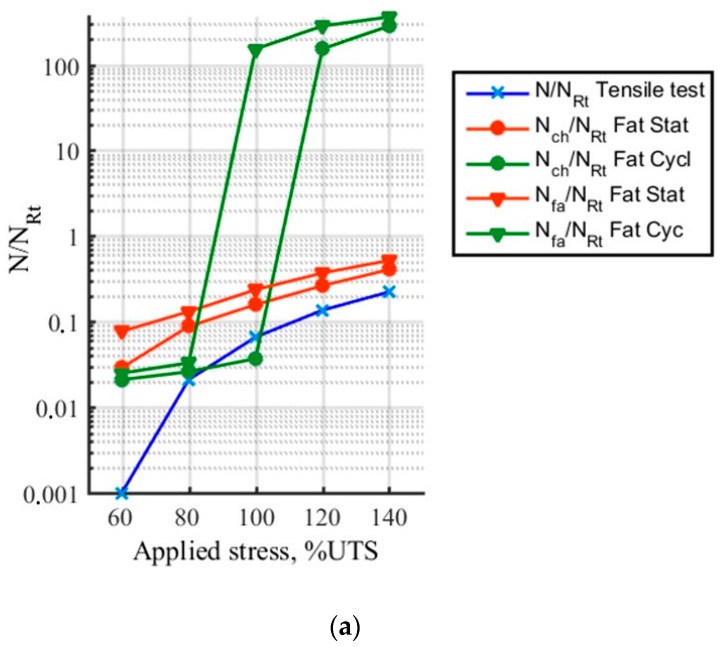

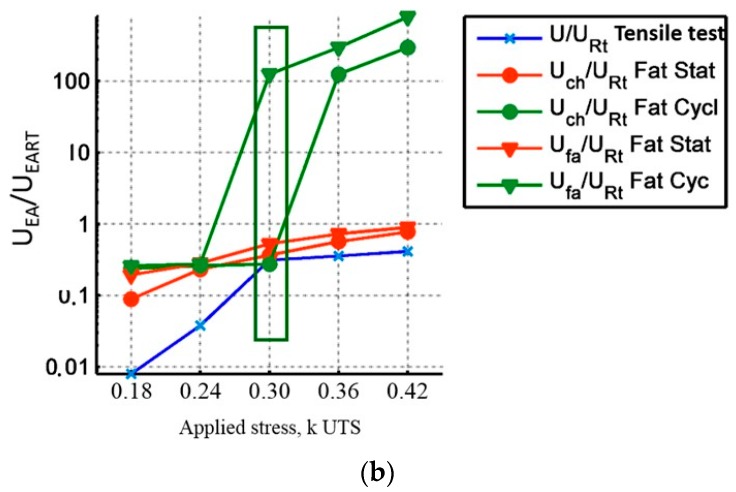
Evolution versus the applied load of (**a**) the ratio N/N_Rt_, (**b**) the ratio U_EA_/U_EART_. The index “ch” means the first loading of each stage and the index ”fa”, the end of the stage.

**Figure 10:**
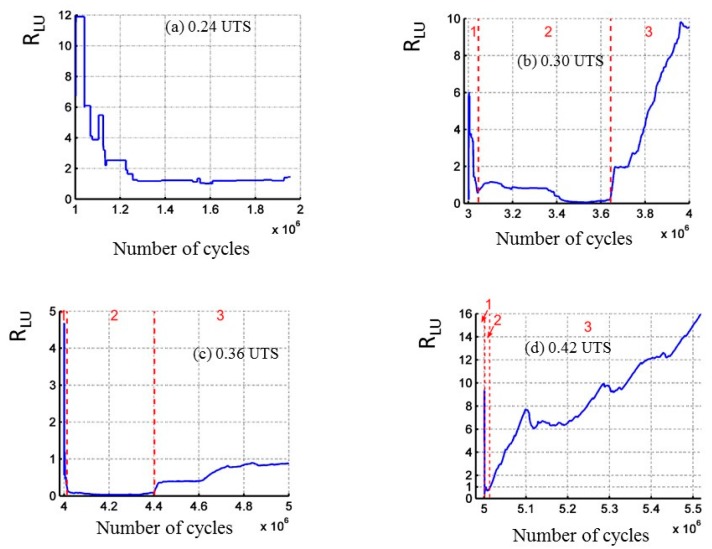
Evolution versus the number of cycles the ratio R_LU_ for differents applied load (**a**) 0.24 UTS; (**b**) 0.30 UTS; (**c**) 0.36 UTS and (**d**) 0.42 UTS.

As regards the acoustic activity in cyclic fatigue test (Figure 9 and Figure 10), a strong increase of the activity is observed from 0.30 UTS, very probably connected to the increase of the frictions at the fiber/matrix and matrix/matrix interfaces. The acoustic activity in static fatigue remains close to that observed in tensile test for these levels of load. Thus it seems that this level of load significantly modifies the behavior of the material, even if it is not still clearly visible on the mechanical parameters. The ratio R_LU_ is calculated for the various levels of load. When the cyclic load has a maximal stress lower than 0.24 UTS, the energy recorded during the loading phase is predominant. This then leads the ratio to be closer to the value 1. From 0.30 UTS, activity may be divided into three phases for each level of load. At first, the ratio R_LU_ is higher than 1. Then the ratio is stable and nearby 1, activity generated during both phases is similar. Finally in the third time, dominant activity during the phases of loading indicates the occurrence of additional damage mechanisms. Furthermore, it should be noted that the second phase identified by this indicator presents a lower duration when the level of load increases. It can be assumed that this level of load is the limit fatigue of this composite. Indeed, it is shown by Kordatos [28] that the strong increase of the acoustic activity is linked to the overtaking of the limit of fatigue. 

### 5.2. Cyclic Fatigue Tests at Maximum Constant Amplitude: Identification of Critical Time

**Figure 11:**
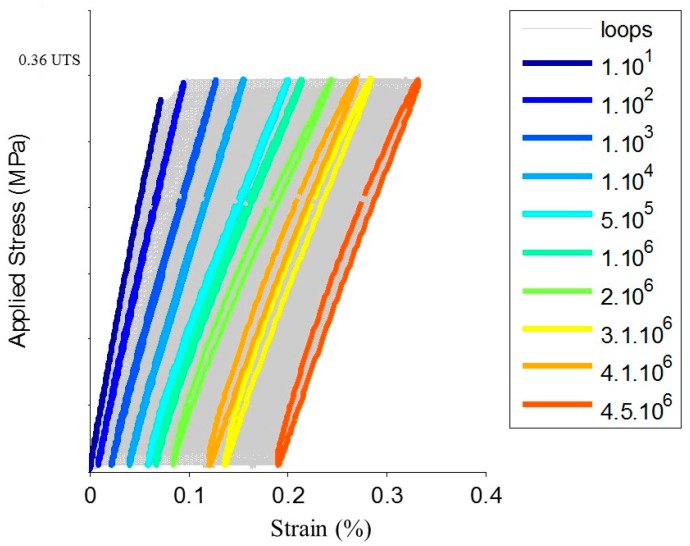
Loops in the plane stress/strain for a cyclic fatigue tests at constant amplitude.

**Figure 12:**
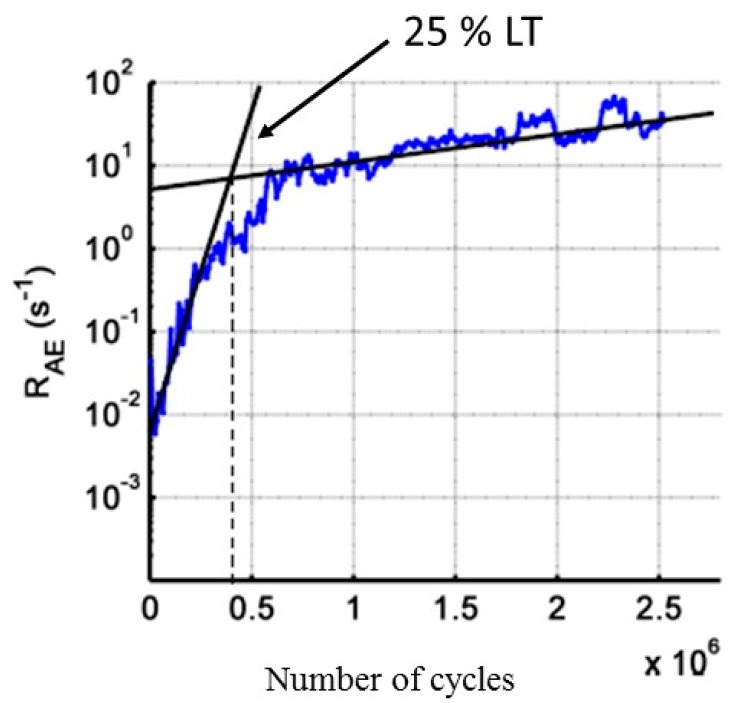
Evolution of the coefficient R_AE_ for cyclic fatigue tests at constant amplitude with the number of cycles.

The cyclic fatigue tests are realized at a constant amplitude just below the fatigue limit (Table 1). The progressive evolution of the hysteresis loops in the plane stress/strain (Figure 11) indicates that the material is damaged from the first cycles of load. This can be explained by the cracking of the matrix in the transverse and longitudinal yarns. Accordingly, the secant elastic modulus decreases continuously from the first cycles of load. The evolution of R_AE_ coefficient versus time is given in Figure 12. For the cyclic fatigue tests at constant amplitude, the evolution of the coefficient R_AE_ is very different from that observed under static fatigue [21]. In both cases for the static fatigue, R_AE_ decreases first, down to a minimum value, and then increases up to the failure of the composite. On average, the minimum of R_AE_ appeared at 55% of the rupture time. The minimum of the coefficient R_AE_ indicates the beginning of the critical damage phase and provides an estimate of the remaining lifetime. In previous studies, the restart of activity prior to final rupture is attributed to the avalanche fibers ruptures. For the cyclic fatigue, a minimum value of the R_AE_ coefficient, is not observed but a significant change of slope is visible for all mechanical tests at approximately 20–25% of the total test duration. 

**Figure 13:**
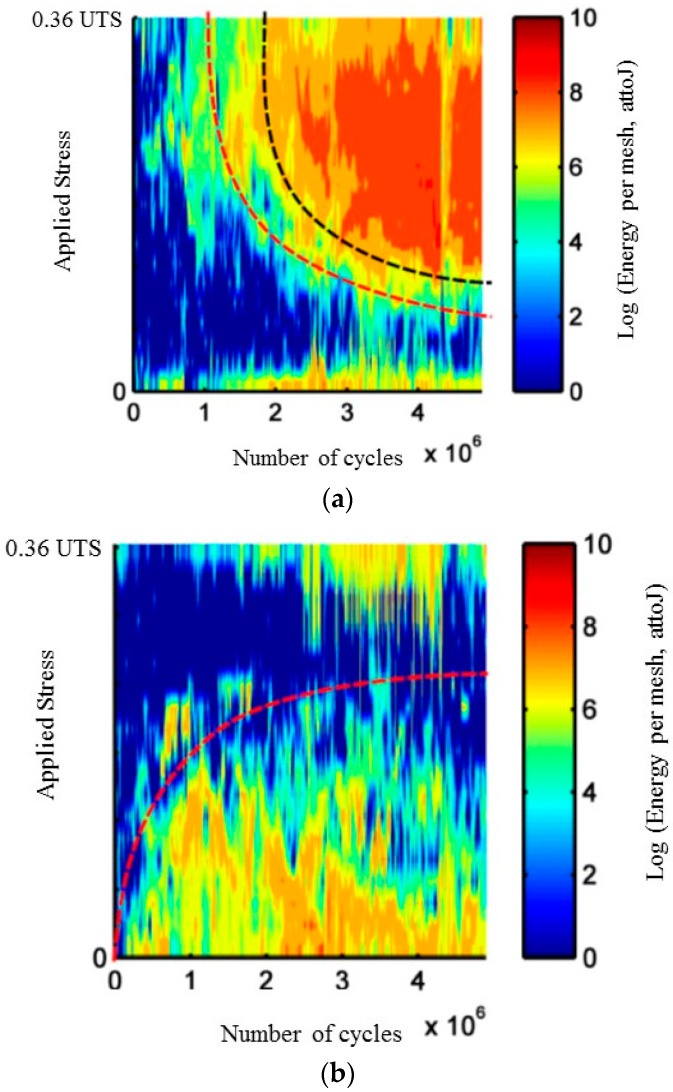
Evolution with the number of cycles of the linear density of acoustic energy recorded during (**a**) loading part and (**b**) unloading part of the cycles on SiC_f_/[Si-B-C] composites for a cyclic fatigue test at constant amplitude 0.36 UTS.

The distribution of the acoustic energy recorded, according to the applied load and the number of cycles during the loading or unloading phases, is represented in Figure 13. The acoustic energy recorded during the first cycles is preferentially located during the loading phase close to the maximal applied load. When acoustic activity intensifies (after a number of cycles about 15% of the test duration) the main part of the acoustic energy is recorded in the neighborhood of *σ*_max._ After, important acoustic activity is recorded for loads lower and lower. On the contrary, the acoustic activity during the unloading phase is registered first of all when the applied load is close to 0, then for more and more raising values of applied load.

Dominant acoustic activity (beyond 40–60% of the life time) during the loading phases, as well as the presence of more and more energy signals, show that an additional mechanism appears in this part of the test. Given that the secant elastic modulus decreases during all the test, meaning a loss of rigidity of the material, while the matrix cracking in the longitudinal yarns takes place rather in the beginning of the test, the main damage mechanism may have changed. The SEM (Scanning electron microscopy) observations showed that debonding between the various layers of matrix in the longitudinal yarns occurs. It is likely that this phenomenon occurs gradually throughout the test, during the phases of loading. The hypothesis according to which the opening of cracks during the phases of loading progressively creates a separation between the longitudinal and transverse yarns may be assumed. 

**Figure 14:**
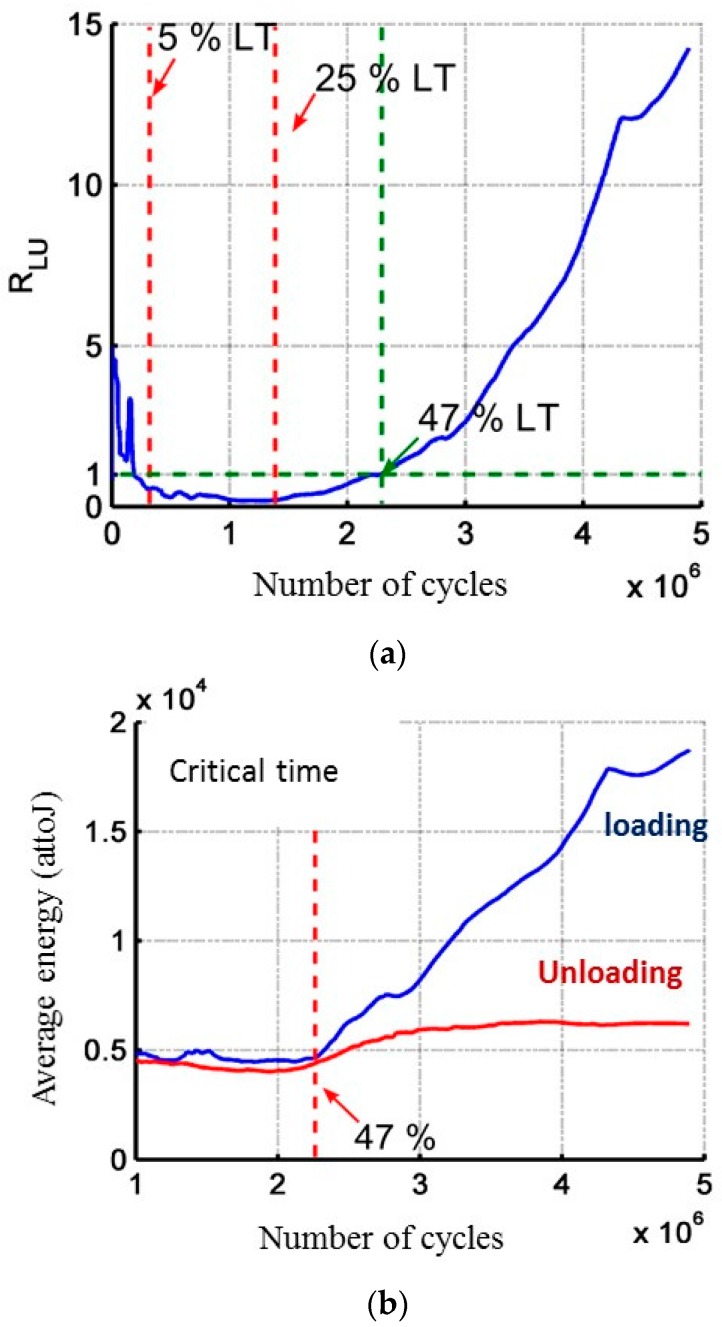
(**a**) Evolution of the coefficient R_LU_ during cyclic fatigue at constant amplitude and highlight of two characteristic times at 25% and 47% of the lifetime duration (LT lifetime) on SiC_f_/[Si-B-C] composites; (**b**) Evolution of the average energy.

The evolution of the coefficient R_LU_ highlights two characteristic times before 50% of rupture time (Figure 14a). This coefficient goes through a minimum around 20–25% of the total test duration and then the coefficient R_LU_ is again up to 1 around 50% of the total test duration. After this characteristic time, the increase of the coefficient R_LU_ is linked to a significant increase in the average energy of the signals recorded during the loading part (Figure 14b), while for the signals recorded during the unloading phases the average energy remains constant. As observed in static fatigue [21], it is possible to define several characteristic times from the AE measurements during cyclic fatigue.

**Figure 15:**
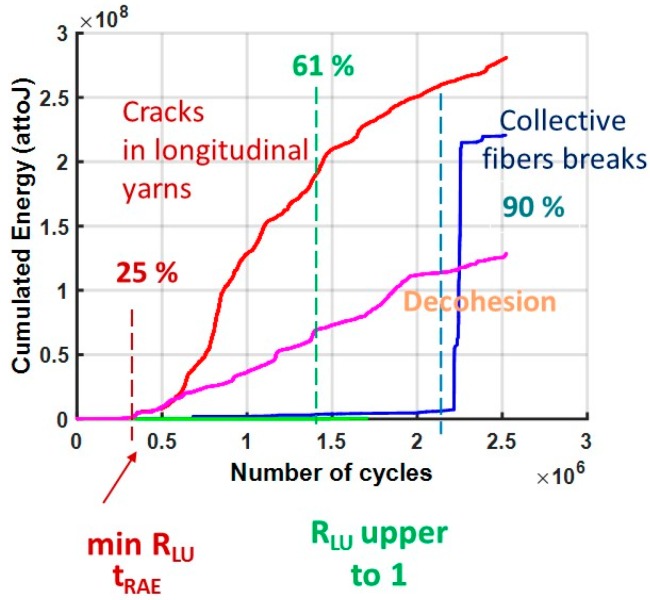
Evolution of damage mechanisms during cyclic fatigue tests at constant amplitude 0.36 UTS. For this test, the characteristics times are at 25% and 61% of the lifetime.

The cluster analysis with RFCAM (Random Forests Classification for Acoustic emission Monitoring) algorithm highlighted various damage mechanisms generated by cyclic loading during the loading phase (Figure 15). First with the cluster E, it is possible to eliminate friction. With the supervised clustering, cluster A is mainly recorded at the end of the test around 90% of the total test duration and the signals are attributed to the breaks of fibers. Cluster B, associated with the matrix cracking in the longitudinal yarns, is significantly activated very early towards 25%. Hence, with this analysis a link is established between the characteristic time at 25% and the beginning of the matrix cracking, but it is not possible with this analysis to make a link with the second characteristic time around 50% and the damage mechanisms. This may be due to the fact that only 50% of the signals are kept after the application of the security voting.

## 6. Conclusions

This paper focuses on the investigation of damage during fatigue tests of CMC at intermediate temperature. Comparison between static and cyclic tests shows that cyclic loading with an amplitude higher than 30% of UTS has significant effects on damage and on material lifetimes. Some new damage indicators are defined based on AE energy activity and dissipated mechanical energy and appear to be more sensitive to damage evaluation than macroscopic mechanical parameters, except internal friction. These new AE indicators show the evolution of damage during long-term tests. The AE method is a powerful method to identify critical times and to predict the remaining lifetime. A link is drawn between a characteristic time at 25% and the beginning of the matrix cracking, in the cyclic fatigue tests. The relationship between the second characteristic time at 50% and identified damage mechanisms requires more investigation at present. Future work will focus on the introduction of new predictive laws beyond this characteristic time, in order to evaluate the remaining lifetime.

## Figures and Tables

**Table 1:** Descriptors of acoustic emission (AE) signals.

Descriptor	Symbol	Unit
Rise Time	RT	µs
Counts	C	-
Duration	D	µs
Amplitude	A	dB
Average Frequency	AF	kHz
Counts to peak	CP	-
Decay Frequency	DF	kHz
Rise Frequency	RF	kHz
Energy	E	attoJ
Rise Time/Duration	RT/D	-
Duration/Amplitude	D/A	µs/dB
Decay time	D-RT	µs
Rise angle	A/RT	dB/µs
Decay Angle	A/(D-RT)	dB/µs
Rise Time/Decay Time	RT/(D-RT)	-
Energy/Amplitude	E/A	attoJ/dB
Counts to peak/Counts	CP/C	-
Amplitude/Frequency	A/AF	dB/kHz
Partial powers PPi f_1_–f_4_	PPi	-
Centroid frequency	FC	kHz
Peak Frequency	PF	kHz

**Table 2:** Results of static and cyclic fatigue tests.

Mechanical Properties	Incremental Static Fatigue (×2)	Incremental Cyclic Fatigue (×3)	Cyclic Fatigue (×3) at Constant Amplitude 0.36 UTS
Stress at failure	84% of UTS	36–42% of UTS	36% of UTS
Strain at failure	*ε*_R_	22–40% of *ε*_R_	12–25% *ε*_R_
Elastic modulus at failure	E_f._v_f_	1.5–2 E_f._v_f_	1.5–2.5 E_f._v_f_
Number of AE sources	9500	2.57 × 10^6^	14 × 10^6^
Cumulated acoustic energy	2.2 × 10^8^ (attoJ)	6.4 × 10^10^ (attoJ)	10 × 10^11^ (attoJ)
matrix cracks spacing/matrix cracks spacing after tensile test	1	1.6–2.4	2
